# Investigating the impact of cigarette smoking behaviours on DNA methylation patterns in adolescence

**DOI:** 10.1093/hmg/ddy316

**Published:** 2018-09-12

**Authors:** Claire Prince, Gemma Hammerton, Amy E Taylor, Emma L Anderson, Nicholas J Timpson, George Davey Smith, Marcus R Munafò, Caroline L Relton, Rebecca C Richmond

**Affiliations:** 1 Medical School, University of Exeter, Exeter, UK; 2 Population Health Sciences, Bristol Medical School, University of Bristol, Bristol, UK; 3 MRC Integrative Epidemiology Unit, University of Bristol, Bristol, UK; 4 Tobacco and Alcohol Research Group, School of Experimental Psychology, University of Bristol, Bristol, UK

## Abstract

Smoking usually begins in adolescence, and early onset of smoking has been linked to increased risk of later life disease. There is a need to better understand the biological impact of cigarette smoking behaviours in adolescence. DNA methylation profiles related to smoking behaviours and cessation in adulthood have been previously identified, but alterations arising from smoking initiation have not been thoroughly investigated. We aimed to investigate DNA methylation in the Avon Longitudinal Study of Parents and Children in relation to (1) different smoking measures, (2) time since smoking initiation and frequency of smoke exposure and (3) latent classes of smoking behaviour. Using 2620 CpG sites previously associated with cigarette smoking, we investigated DNA methylation change in relation to own smoking measures, smoke exposure duration and frequency, and using longitudinal latent class analysis of different smoking behaviour patterns in 968 adolescents. Eleven CpG sites located in seven gene regions were differentially methylated in relation to smoking in adolescence. While only *AHRR* (cg05575921) showed a robust pattern of methylation in relation to weekly smoking, several CpGs showed differences in methylation among individuals who had tried smoking compared with non-smokers. In relation to smoke exposure duration and frequency, cg05575921 showed a strong dose–response relationship, while there was evidence for more immediate methylation change at other sites. Our findings illustrate the impact of cigarette smoking behaviours on DNA methylation at some smoking-responsive CpG sites, even among individuals with a short smoking history.

## Introduction

Smoking commonly begins in adolescence (1), with 19% of adolescents (11- to 15-year-olds) in the UK having ever smoked in 2016 (2). While this percentage has been decreasing, from 49% in 1996 (3), early onset of smoking has been linked to an increased risk of cancer (4,5) and other diseases (6,7) in later life. Although the harms increase with increased smoking intensity and duration, even light smokers are at increased risk compared with people who don’t smoke (8). Consequently, there is a need to understand the biological impact of cigarette smoking behaviours in adolescence. Findings may also allow a more informed education to be given to adolescence concerning the harms of any amount of smoking.

Cigarette smoking has been associated with DNA methylation in several recent epigenome-wide association studies (EWAS) (9–16). This differential methylation in relation to smoke exposure has also been linked to disease, including a number of epithelial cancers (17,18), and therefore could be used as a potential biomarker of both smoking status, length of smoking history and in turn as a predictor of disease risk (9,17). However, site-specific DNA methylation in response to smoking is dynamic and can change over time. Although several studies have investigated the extent to which changes in DNA methylation that are associated with smoking persist after smoking cessation (10,19,20), the length of time required for smoking to impact on DNA methylation has not been fully evaluated.

Several studies have shown that smoking is associated with site-specific DNA methylation among young people and adolescents (12,21,22), suggesting that methylation changes may be induced after a relatively short smoking history. However, these studies have typically evaluated methylation at only a few CpG sites and have not modelled methylation changes with time since smoking initiation or considered the impact of light or occasional smoking on DNA methylation.

Furthermore, previous studies have not thoroughly considered the potential confounding effect of maternal smoking in pregnancy on associations between own smoking and DNA methylation in adolescence, which is important given the strong overlap in DNA methylation profiles among smokers and those exposed to smoke *in utero*, and the persistent impact of prenatal smoke exposure on DNA methylation at some CpG sites across the lifecourse (23). In addition, it is of interest to evaluate whether adolescents exposed to smoke *in utero* are more susceptible to further change in DNA methylation levels at candidate CpG sites when they initiate smoking compared to those not exposed *in utero*. This work aimed to investigate DNA methylation measured by the Illumina Infinium® HumanMethylation450 (HM450) BeadChip in the Avon Longitudinal Study of Parents and Children (ALSPAC) adolescents (median age 17 years 7 months; hereafter referred to as 17 years) in relation to (1) different smoking exposures (ever smoking, current weekly smoking, ever weekly smoking and blood cotinine levels), (2) time since smoking initiation and frequency of smoke exposure and (3) several latent classes of smoking behaviour (non-smoker, experimenters, late-onset regular smokers and early-onset regular smokers) (24).

## Results

### Study sample

**Figure 1:**
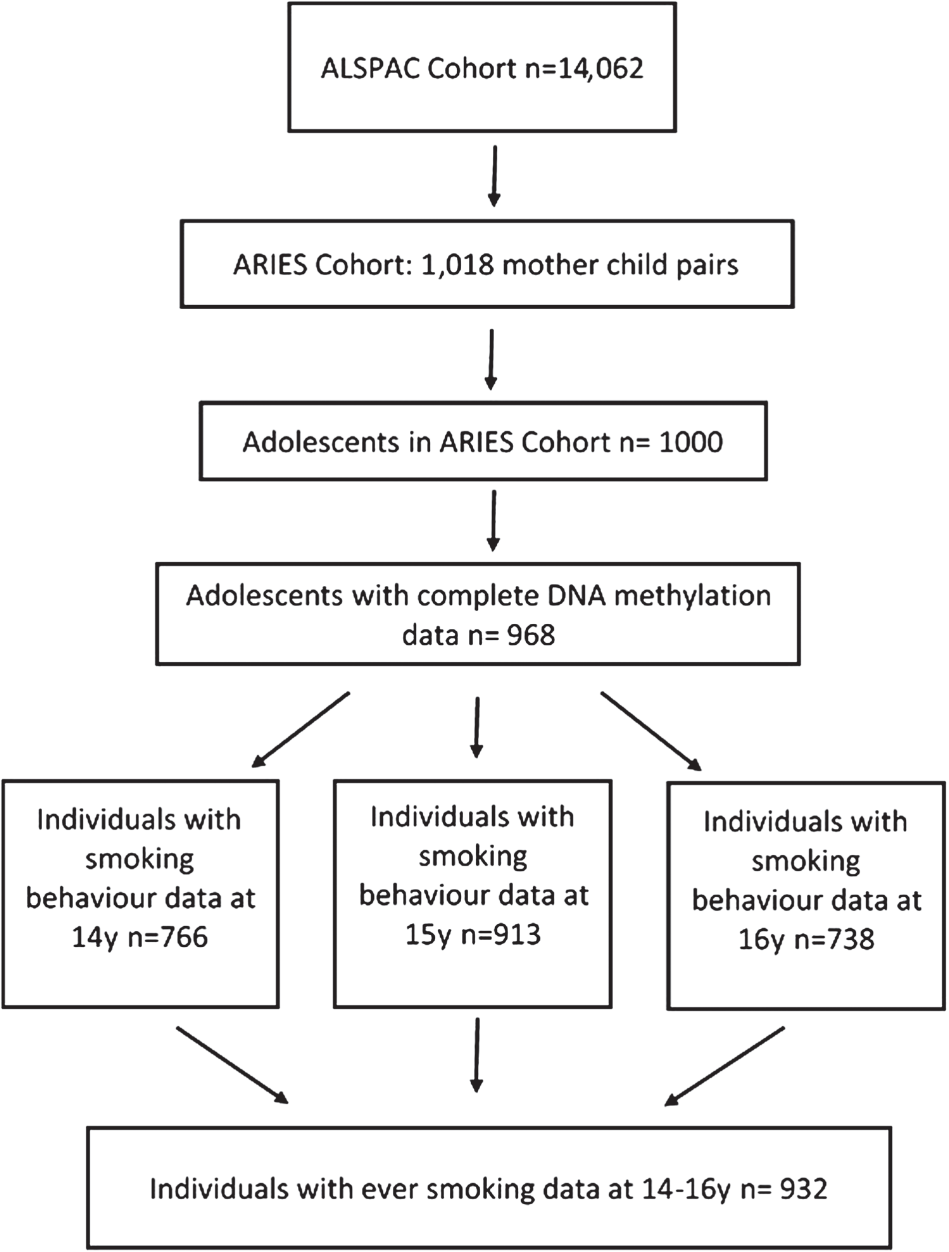
Participant flow diagram illustrating participants included in this analysis.

A total of 968 individuals in the Accessible Resource for Integrated Epigenomics Studies (ARIES) subsample of ALSPAC had DNA methylation derived from samples taken in adolescence (mean age 17 years) (25). Information on ever smoking across all three questionnaires (ages 14, 15 and 16) was available for 932 individuals (Fig. 1). Of the 834 individuals with information on current weekly smoking, 73 were classified as weekly smokers while 761 were classified as less than weekly smokers at age 16. Individuals who were weekly smokers in ARIES were more likely to be female, to be slightly younger, to regularly use cannabis, to drink regularly and to have used substances than non-weekly smokers and were more likely to have been exposed to maternal smoking during pregnancy (Table 1). Comparisons between participants based on the other smoking definitions and latent classes are shown in Table S1.

**Table 1:** Baseline characteristics of ARIES subsample of adolescents from the ALSPAC by current weekly smoking status

Variable	ARIES (n = 968)	Current weekly smokers (n = 73)	Nonweekly smokers (n = 761)	*P*-value difference
Sex (% Male) (n)	48.2 (467)	21.9 (16)	48.8 (371)	<0.001
Passive smoke exposure (% yes) (n)	49.3 (477)	58.9 (43)	43.1 (328)	0.009
Age at DNA methylation collection [mean (SD)]	**(n = 968)**	**(n = 71)**	**(n = 737)**	
	17.1 (1.1)	17.0 (1.2)	17.2(1.0)	0.111
Maternal smoking during pregnancy (% yes) (n)	**(n = 945)**	**(n = 66)**	**(n = 725)**	
	10.3 (97)	22.7 (15)	8.1 (59)	<0.001
Manual occupations^a^ [% (n)]	**(n = 879)**	**(n = 60)**	**(n = 684)**	
	14.2 (125)	21.7 (13)	13.0 (89)	0.062
Regular alcohol consumption (%yes) (n)	**(n = 964)**	**(n = 73)**	**(n = 752)**	
	50.4 (486)	79.4 (58)	48.9 (368)	<0.001
Substance use	**(n = 968)**	**(n = 73)**	**(n = 761)**	
Regular cannabis use (%yes) (n)	5.2 (50)	27.4 (20)	1.2 (9)	<0.001
Any substance use (%yes) (n)	43.4 (420)	89.0 (65)	34.0 (259)	<0.001

Missing current weekly smoking data (n = 134).

^a^Based on the 1991 OPCS classification.

### Multivariable linear regression with own smoking behaviours

Using information on ever smoking and ever weekly smoking derived from questionnaires obtained at ages 14–16, as well as current weekly smoking and blood cotinine levels obtained at age 16, multivariable linear regression was used to identify CpG sites associated with smoke exposure in adolescence. Specifically, we investigated methylation β values for 2620 out of 2623 CpGs previously identified in a large-scale EWAS of smoking (10) which were available in the normalized dataset for the ARIES offspring in adolescence (mean age 17 years). CpG sites identified in a basic multivariable linear regression model adjusted for batch and cell count were investigated in additional regression models with adjustment for potential confounders. Due to missing covariate data, multiple imputation (MI) of covariates was carried for the adjusted models (as outlined in Table S2).

#### Ever smoking

For ever smoking, four CpGs passed the Bonferroni-corrected threshold in the basic model; cg19593285 *(E2F1*), cg02512902 *(KSR1),* cg03519879 *(C14ORF43)* and cg13951797 *(TRAF7)*. For these sites, there was a reduction in methylation in relation to ever smoking (ranging from 1.1 and 1.4% in the Adjusted Model 1) which showed some degree of attenuation with further covariate adjustment (ranging from 0.7 to 1.2% in the Adjusted Model 3). Sites cg19593285 *(E2F1*), cg02512902 *(KSR1)* and cg13951797 *(TRAF7)* showed large attenuation of effects in the fully adjusted model and this variable showed the highest degree of attenuation with covariate adjustment (Table 2).

**Table 2:** Multivariable linear regression of smoking behaviours and DNA methylation with top sites from basic model taken forward to models 1–3 after imputation

	Basic model			Adjusted model 1			Adjusted model 2			Adjusted model 3			Adjusted model 4		
Ever smoked a cigarette reliable (n = 504 ever; n = 435 never)															
Total sample size	910														
cg02512902 *(KSR1*)	−0.016	−0.023,	3.64e-06	−0.014	−0.021,	6.36e-05	−0.012	−0.019,	0.002	−0.011	−0.018,	0.003	−0.011	−0.019,	0.002
		−0.009			−0.007			−0.004		−0.011	−0.004			−0.004	
cg19593285 *(E2F1)*	−0.015	−0.021,	1.83e-05	−0.012	−0.018,	1.13e-04	−0.012	−0.018,	3.30e-04	−0.012	−0.016,	2.44e-04	−0.008	−0.015,	0.029
		−0.008			−0.006			−0.005			−0.006			−0.001	
cg13951797 (*TRAF7*)	−0.016	−0.024,	1.34e-05	−0.011	−0.018,	0.003	−0.008	−0.016,	0.033	−0.007	−0.015,	0.065	−0.009	−0.016,	0.025
		−0.009			−0.004			−0.001			4.46e-04			−0.001	
cg03519879 *(C14orf43)*	−0.013	−0.019,	1.80e-05	−0.014	−0.021,	6.36e-05	−0.012	−0.019,	0.002	−0.011	−0.018,	0.003	−0.012	−0.018,	3.93e-04
		−0.007			−0.007			−0.004			−0.004			-0.005	
Current weekly smoking (n = 71 yes; n = 762 no)															
Total sample size	808														
cg05575921 *(AHRR)*	−0.040	−0.051,	3.10e-14	−0.038	−0.048,	5.17e-13	−0.036	−0.047,	9.39e-10	−0.035	−0.046,	7.56e-10	−0.033	−0.046,	7.35e-09
		−0.030			−0.027			−0.024			−0.024			−0.022	
Ever smoked weekly (n = 132 ever; n = 848 never)															
Total sample size	949														
Cg05575921 (*AHRR)*	−0.035	−0.043,	5.90e-17	−0.032	−0.024,	8.88e-15	−0.028	−0.037,	1.38e-09	−0.028	−0.037,	4.05e-10	−0.028	−0.037,	4.85e-10
		−0.027			−0.040			−0.019			−0.019			−0.019	
CG08331398 (*PSMB8)*	−0.020	−0.028,	4.88e-06	−0.019	−0.010,	2.17e-05	−0.022	−0.031,	1.36e-05	−0.021	−0.031,	1.25e-05	−0.020	−0.030,	3.84e-05
		−0.011			−0.027			−0.012			−0.012			−0.011	
Cotinine in the blood 10 ng/ml															
Total sample size	770														
CG05575921 (*AHRR*)	−0.004	−0.005,	4.19e-16	−0.004	−0.005,	3.24e-13	−0.003	−0.004,	6.01e-10	−0.003	−0.004,	2.79e-10	−0.003	−0.004,	1.41e-10
		−0.003			−0.003			−0.002			−0.002			−0.002	
CG06338710 (*GFI1*)	−0.006	−0.008.,	1.73e-06	−0.006	−0.008,	2.12e-06	−0.006	−0.008,	2.91e-05	−0.004	−0.007	3.67e-04	−0.004	−0.006,	7.30e-04
		−0.003			−0.003			−0.003			−0.002			−0.001	
CG09935388 (*GFI1*)	−0.007	−0.009,	2.98e-08	−0.006	−0.009,	2.35e-07	−0.006	−0.009,	1.15e-06	−0.004	−0.005,	5.67e-05	−0.005	−0.007,	4.86e-07
		−0.004			−0.004			−0.004			−0.002			−0.003	
CG12876356 (*GFI1*)	−0.007	−0.010,	1.74e-06	−0.007	−0.010,	4.09e-06	−0.007	−0.010,	2.01e-05	−0.004	−0.006,	4.29e-04	−0.005	−0.007,	2.81e-04
		−0.004			−0.004			−0.004			−0.002			−0.002	
CG18146737 (*GFI1*)	−0.006	−0.008,	1.29e-05	−0.006	−0.009,	2.21e-05	−0.006	−0.009,	4.19e-05	−0.002	−0.003,	0.002	−0.004	−0.006,	9.54e-04
		−0.003			−0.003			−0.003			−7.47e-04			−0.001	
CG26703534 (*AHRR*)	−0.003	−0.004,	3.11e-07	−0.003	−0.004,	2.04e-06	−0.002	−0.004,	1.17e-05	−0.002	−0.003,	1.19e-05	−0.002	−0.004,	1.26e-05
		−0.002			−0.001			−0.001			−7.47e-04			−0.001	

Basic model: Adjusted model for batch and cell count. Adjusted model 1: Adjusted model for batch, cell count, age, sex, social class and maternal smoking during pregnancy. Adjusted model 2: Adjusted model for batch, cell count, age, sex, social class, regular cannabis use, regular alcohol use, passive smoke and maternal smoking during pregnancy. Adjusted model 3: Adjusted model for batch, cell count, age, sex, social class, regular cannabis use, regular alcohol use, passive smoke, maternal smoking during pregnancy and methylation at age 7. Adjusted model 4: Adjusted model for batch, cell count, age, sex, social class, regular cannabis use, regular alcohol use, passive smoke, maternal smoking during pregnancy and cord blood methylation.

Bonferroni threshold = 1.91e-05.

#### Current weekly smoking

Cg05575921 (*AHRR*) was the only CpG site to pass the Bonferroni-corrected threshold with current weekly smoking in all regression models. Adjusted Model 1 showed a methylation change of −3.8% between current weekly smokers and non-weekly smokers (β = −0.038 (95% CI −0.048, −0.027; *P* = 5.17x10^−13^), which remained largely unchanged after additional covariate adjustment (β = −0.033 (95% CI -0.046, −0.024, P = 7.56x10^−10^) in the fully adjusted model (Adjusted Model 3) (Table 2).

#### Ever weekly smoking

Cg05575921 (*AHRR*) and cg08331398 *(PSMB8*) passed the Bonferroni-corrected threshold in relation to ever weekly smoking in all regression models. In Adjusted Model 1, for the *AHRR* site there was a methylation change of −3.2% between ever weekly smokers and never weekly smokers (β = −0.032 (95% −0.040, −0.024; P = 8.88x10^−15^), which remained largely unchanged after additional covariate adjustment (β = −0.028 (95% CI −0.037, −0.019; *P* = 4.05x10^−10^) in the fully adjusted model (Adjusted Model 3)). In Adjusted Model 1, for *PSMB8* there was a methylation change of −1.9% between ever weekly smokers and never weekly smokers (β = −0.019 (95% CI −0.027, −0.010; P = 2.17x10^−5^), which also remained largely unchanged after additional covariate adjustment (β = −0.021 (95% CI −0.031, −0.012; *P* = 1.25x10^−5^) in Adjusted Model 3 (Table 2).

#### Blood cotinine levels

Six sites passed the Bonferroni-corrected threshold in the basic model in relation to continuous blood cotinine levels; cg05575921 (*AHRR*), cg06338710 (*GFI1*), cg09935388 (*GFI1*), cg12876356 (*GFI1*), cg18146737 (*GFI1*) and cg26703534 (*AHRR*). For all sites, there was a reduction in methylation (between −0.007 and −0.003 per 10 ng/ml) in Adjusted Model 1, which attenuated slightly in the fully adjusted model (Adjusted Model 3) (between −0.004 and −0.002 per 10 ng/ml) (Table 2).

### Complete case sensitivity analysis

For the own smoking analysis, multivariable linear regression was also performed using the complete cases for each model. The results of the complete case analysis are largely consistent with respect to effect estimates although *P*-values were larger for the adjusted models (largely due to smaller sample size) (Table S3).

### Time since initiation and frequency of smoke exposure

**Figure 2:**
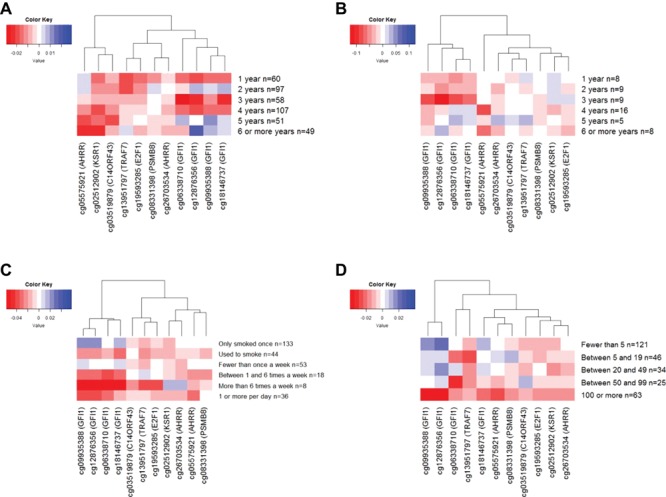
(**A**) Difference in methylation between time since individuals first ever smoked and never smokers. Adjusted for batch effect, cell count, age, sex, social class and maternal smoking during pregnancy. (**B**) Difference in methylation between time since individuals started weekly smoking and never smokers. Adjusted for batch effect, cell count, age, sex, social class and maternal smoking during pregnancy. (**C**) Difference in methylation between smoking intensity and never smokers. Adjusted for batch effect, cell count, age, sex, social class and maternal smoking during pregnancy. (**D**) Difference in methylation between number of cigarettes ever smoked and never smokers. Adjusted for batch effect, cell count, age, sex, social class and maternal smoking during pregnancy.

We next investigated the patterns of methylation change with years since individuals first smoked, years since individuals started weekly smoking and intensity of smoke exposure. CpG sites which passed the Bonferroni threshold in the basic model of the multivariable linear regression analysis (Table 2) were taken forward for this analysis. Figures 2A–2D shows the β differences in methylation of the 11 CpG sites identified in the own smoking linear regression compared with methylation in the relevant non-smoking category for each variable, adjusted for batch effect, cell count, age, sex, social class and maternal smoking during pregnancy.

Several of the CpGs showed strong differences in methylation in relation to the time since the individual first ever smoked (Fig. 2A), with evidence of a dose–response for some sites [cg05575921 (*AHRR*) and cg02512902 *(KSR1)*] and a more immediate impact of smoking on others [cg13951797 (*TRAF7)* and cg19593285 (*E2F1)*]. Cg05575921 (*AHRR*) showed a similar dose–response pattern in relation to time since starting weekly smoking, while sites in *GFI1* (cg09935388, cg12876356, cg06338710 and cg18146737) showed a more immediate impact of weekly smoking (Fig. 2B). In relation to intensity of smoke exposure, cg05575921 (*AHRR*) again showed a strong dose–response, with decreasing levels of methylation in relation to intensity of smoking behaviours and number of cigarettes smoked (Fig. 2C and 2D). On the other hand, there was a more immediate methylation change at cg13951797 *(TRAF4),* cg02512902 *(KSR1)* and cg19593285 *(E2F1)*, which was largely maintained as the number of cigarettes smoked increased (Fig. 2D).

### Longitudinal latent class analysis

Using repeated measures of smoking frequency obtained from questionnaire data at 14, 15 and 16 years, longitudinal latent class analysis (LLCA) was performed using *Mplus v8* (26), as has been described previously (24). A 4-class model was selected for the repeated measures of smoking frequency (see Table S4 for model fit statistics). This 4-class solution comprised smoking behaviour patterns that we refer to as never-smokers (86%), experimenters (5%), late onset regular smokers (9%) and early onset regular smokers (1%). These classes are consistent with those derived previously by Heron and colleagues (24), where non-smokers reported very little or no smoking, experimenters smoked infrequently (monthly), late-onset regular smokers were individuals who began smoking by age 14 and were mostly daily smokers by age 16 and early-onset regular smokers were mostly daily smokers by age 14. Figure S1 shows the within-class probabilities for the repeated measures of smoking frequency. Given the very high entropy of the 4-class model (0.94), individuals were assigned to the class for which they had the highest probability of class membership (modal class assignment) and the latent classes were used as an observed categorical exposure in further analyses.

CpG sites that passed the Bonferroni-corrected threshold in the basic model of the linear regression analysis were taken forward for the LLCA. Three of the 11 identified CpG sites from the linear regression analyses passed the Bonferroni-corrected threshold in the association with the latent classes [p = 0.0045 (0.05/11)]: cg05575921 (*AHRR*), identified in relation to total number of cigarettes smoked and current smoking, cg08331398 (*PSMB8*), which was identified in relation to individuals who had ever smoked weekly and cg26703534 (*AHRR*), identified in the continuous blood cotinine levels analysis. Cg05575921 (*AHRR)* was found to be differentially methylated across the latent classes (*P* = 5.3 × 10^−10^) in a dose–response manner, whereby early onset regular smokers had a 5.4% (95% CI −2.8, −8.1) reduction in methylation compared with never smokers, late onset smokers had a 2.5% (−1.7, −3.4) reduction in methylation and experimenters have a 0.1% (−1.3, 1.1) reduction in methylation. Methylation at *PSMB8* (*P* = 0.0026) was most strongly associated with late onset (versus never) smoking, where late onset smokers had a 1.7% reduction in methylation compared with never smokers (95% CI 0.8, 2.6). Cg26703534 (*AHRR*) was also found to be differentially methylated across the latent classes (*P* = 7.0 × 10^−4^) in a dose–response manner, whereby early onset regular smokers had a 5.2% (95% CI −7.9, −2.4) reduction in methylation compared with never smokers, late onset smokers had a 0.9% (−1.8, −4.6x10^−5^) reduction in methylation and experimenters have a 0.6% (−1.8, 0.6) reduction in methylation (Table 3).

**Table 3:** Latent class analysis for the 11 CpGs identified in linear regression analysis


	**Overall (n = 931)**	**Early onset (n = 8) versus never (n = 800)**		**Late onset (n = 80) versus never (n = 800)**		**Experimenter (n = 43) versus never (n = 800)**	
CpG site	***P*-value of f-test for model fit**	***β***	**95% CI**	***β***	**95%CI**	***β***	**95% CI**
cg05575921 (*AHRR)*	5.32E-10	−0.054	−0.081, −0.028	−0.025	−0.034, −0.017	−0.001	−0.013, 0.011
cg26703534 (*AHRR*)	6.98E-04	−0.052	−0.079, −0.024	−0.009	−0.018, −4.57E-05	−0.006	−0.018, 0.006
cg08331398 (*PSMB8*)	2.65E-03	0.012	−0.016, 0.040	−0.017	−0.026, −0.008	−0.007	−0.020, 0.005
cg09935388 (*GFI1*)	0.019	−0.039	−0.100, 0.023	−0.030	−0.050, −0.010	0.004	−0.023, 0.031
cg02512902 (*KSR1)*	0.021	−0.004	−0.035, 0.028	−0.012	−0.023, −0.002	−0.016	−0.030, −0.002
cg03519879 (*C14orf43)*	0.023	0.011	−0.018, 0.040	−0.013	−0.022, −0.003	−0.010	−0.023, 0.002
cg06338710 (*GFI1*)	0.042	−0.043	−0.107, 0.021	−0.028	−0.049, −0.049	−0.007	−0.035, 0.021
cg12876356 (*GFI1*)	0.045	−0.028	−0.095, 0.039	−0.031	−0.053, −0.009	0.002	−0.028, 0.031
cg18146737 (*GFI1*)	0.069	−0.001	−0.080, 0.077	−0.034	−0.060, −0.008	0.010	−0.024, 0.045
cg13951797 *(TRAF7)*	0.248	−0.028	−0.063, 0.007	−0.005	−0.017, 0.006	−0.009	−0.024, 0.007
cg19593285 *(E2F1)*	0.954	0.007	−0.025, 0.039	−0.001	−0.012, 0.009	−0.002	−0.016, 0.012
							

Models adjusted for batch, cell count, age, sex, social class and maternal smoking in pregnancy. Bonferroni threshold: 0.0045.

### Interaction analysis

The interaction between the four own smoking measures: ever tried a cigarette, current weekly smoker, ever smoked on a weekly basis and blood cotinine levels and maternal smoking during pregnancy was tested on 119 CpG sites found to be associated with both own (10) and maternal smoking during pregnancy (27) in two large EWAS. One CpG site passed the Bonferroni-corrected threshold for interaction with cotinine: cg06338710 (*GFI1*) (*P* = 3.86 × 10^−4^) (full results in Table S5), where there was evidence for a greater reduction in DNA methylation in response to cotinine among adolescents exposed to maternal smoking in pregnancy than those who weren’t (Fig. S2).

### Cell counts

To investigate the potential role of cell sub-composition in mediating the impact of smoke exposure on DNA methylation, we investigated associations between the smoking behaviours and cell-type fraction estimated using the methylation data (28,29). Ever smoking and current weekly smoking in adolescence were not strongly associated with cell-type fractions, with the exception of CD4 T cell count which was higher among currently weekly smokers compared with non-smokers at age 17 (difference in proportion = 0.018 (95% CI 0.006, 0.031; p = 0.005 in model adjusted for age 7 cell counts), which is consistent with previous findings (30) (Table S6). However, all analyses investigated adjustment for derived cell count proportions, which were not generally shown to attenuate results.

## Discussion

We investigated methylation at 2620 CpG sites previously associated with smoking in a large-scale EWAS (10) in relation to smoking behaviours of a cohort of adolescents. Eleven CpG sites showed robust associations with various smoking behaviours: ever smoking, current weekly smoking, ever weekly smoking and blood cotinine levels. Furthermore, the associations we observed were largely supported by analysis of methylation in relation to duration and frequency of smoke exposure, and in LLCA which was used to group adolescents based on their smoking habits.

Cg05575921 (*AHRR*) showed the strongest degree of differential methylation with current smoking but did not appear in ever smoking analysis, suggesting that a longer and sustained smoking history is required for this site to become differentially methylated. This is supported by the finding of a dose–response in methylation at this site in relation to both duration and intensity of smoke exposure. Furthermore, both cg05575921 and cg26703534, also annotated to *AHRR*, were found to be strongly associated with blood cotinine levels and in the relation to early onset regular smoking in the latent class analysis.

While other studies have suggested that only a short smoking history is required for cg05575921 (*AHRR*) to become differentially methylated (15,31), these studies have not outlined the exact time frame required for this to become established. Results of our study suggest that hypomethylation at this site becomes apparent with ≥4 years smoke exposure, and with smoking at least once a week. This is consistent with the finding by Philibert *et al.* (12), where associations were observed with less than one-half pack year of smoke exposure. Cg05575921 and cg26703534 are situated in intron 3 of the aryl hydrocarbon receptor repressor (*AHRR*) gene on chromosome 5. Cg05575921 has been previously identified in relation to smoke exposure in several EWAS and is frequently found to be the CpG site most strongly associated with exposure (10,15,21,31,32). Cg05575921 (*AHRR*) methylation has also been identified as a potential biomarker for lung cancer (33) and subclinical atherosclerosis in smokers (32).

Cg02512902 *(KSR1)* and cg03519879 (*C14ORF43*) were consistently associated with ever smoking and showed evidence for differential methylation with a small degree of smoke exposure, which was then maintained with increased duration of smoking. There was also evidence for an association between *KSR1* and C14ORF43 methylation and the latent classes, with the largest degree of differential methylation among the late onset smokers and experimenters compared with non-smokers. Cg08331398 (*PSMB8*) was strongly associated with ever weekly smoking in all linear regression models and also with late onset smoking in the latent class analysis.

Cg02512902 maps to the Kinase Suppressor of Ras 1 (*KSR1*) gene which transcribes proteins that positively regulate the Ras signalling pathway (34). It has been associated with cancer and identified as a potential therapeutic target (35). Cg03519879 maps to the *C14ORF43* gene, synonymous with *ELMSAN1*, which has not been researched considerably but is thought to play a role in chromatin binding. Cg08331398 maps to the *PSMB8* gene and has been associated with several types of cancer (36–39) and hepatitis B (40,41).

Sites in *GFI1* (cg09935388, cg12876356, cg06338710 and cg18146737) were identified in the analysis of blood cotinine levels and there was evidence for a more immediate impact of weekly smoking on these sites. The growth factor independent 1 transcriptional repressor gene (*GFI1*) is most highly expressed in the bone marrow and has been associated with immune system disruption, and conditions such as haematopoiesis, neutropenia, leukaemia and prostate cancer among others (42–46). At *GFI1* (cg06338710) there was also some evidence for an interaction between own smoking and maternal smoking in adolescence.

A key strength of this study is the longitudinal cohort design, which enabled an evaluation of DNA methylation in relation to smoking behaviour reported at multiple time points in adolescence. The quantity of smoking data obtained from the questionnaires, which captured different elements of smoking behaviour, enabled an evaluation of different types of smoking behaviour. Use of multiple time points allowed us to derive a more reliable measure of ever smoking, to explore the dynamics of methylation in relation to time since smoking initiation (first trying and first regularly smoking) and smoking intensity and permitted the LLCA. Furthermore, as DNA methylation data at birth and age 7 were available, adjustment for baseline levels of methylation in later models ameliorated risk of reverse causality (i.e. where methylation influenced smoking behaviour rather than vice versa). The wealth of phenotypic data in ALSPAC has aided a thorough assessment of potential confounding factors, and MI was used to account for missing data in order to fully evaluate this.

The first limitation of the analysis is that the ARIES cohort represents a highly selected sample of participants who are not representative of the wider ALSPAC cohort (25). This is illustrated with the small number of smokers in this adolescent cohort, with only 73 people (10%) identified as at least weekly smokers at age 16, compared with an estimated 15% among individuals who provided partial smoking information in the wider ALSPAC cohort (24). While we attempted to maximize power in this study by only focusing on those CpGs which have been previously identified as being responsive to tobacco smoke (10), potential selection bias in this setting remains an issue (47). The second limitation is that some individuals inconsistently reported their smoking behaviour across the three-time points, which reduced the reliability of the smoking data. Although we derived a variable for ever smoking to account for this inconsistency, across all the own smoking variables there may have been more unreliable responses that could have affected the results. Thirdly, differential measurement error of mothers’ reporting of her own smoking behaviour during pregnancy could also have biased associations. Fourthly, this analysis was limited to blood samples with mixed cell composition. While models were adjusted for derived cell counts and the analysis was based on CpG sites with robust associations with smoke exposure, which were not driven by cellular heterogeneity, the limitation of tissue specificity as well as the lack of expression data currently available on these samples limits the assessment of functional consequences of these methylation changes. However, functional annotation of the differentially methylated CpG sites suggests they are involved in important pathways.

The results of this study illustrate the impact of cigarette smoking on DNA methylation at some smoking-responsive CpG sites even with a relatively limited smoking history. This study supports previous evidence that methylation at cg05575921 (*AHRR*) could serve as an important biomarker for smoke exposure among adolescents (21), especially among those with a longer smoking history and increases in a dose–response manner in relation to both time since initiation and frequency of smoke exposure. This study also implicates other CpG sites that could serve as biomarkers of even shorter smoking history, being evident among both late onset smokers and experimenters. Although these findings were supported in multiple models, replication of associations at these CpG sites in an independent cohort is required. Further research is also required to investigate the recovery of DNA methylation at sites responsive to smoking within adolescence, especially if these individuals do not go on to smoke regularly in adulthood. Four of the 11 CpG sites identified here were identified in a study investigating DNA methylation change in response to smoking cessation (20): cg19593285 (*E2F1*), cg03519879 (*C14ORF43*), cg05575921 (*AHRR*) and cg26703534 (*AHRR*). Of these four, DNA methylation changes were found to persist for between 11 and 47 years after smoking cessation. However, as this study investigated smoking cessation among a cohort of adults who had likely had a much longer history of smoke exposure, we cannot reliably infer a link between smoking habits in adolescence and persistence in DNA methylation. Therefore, future research is needed to investigate long-term patterns of DNA methylation in individuals who smoked during adolescence but not into adulthood. Finally, we advocate the need for further downstream analyses to investigate potential causal mechanisms by which DNA methylation may mediate the impact of early life smoke exposure on later life disease risk.

## Materials and Methods

### Study participants

The ALSPAC is a large, prospective cohort study based in the South West of England (48,49). A total of 14 541 pregnant women resident in Avon, UK, with expected dates of delivery April 1, 1991 to December 31, 1992 were recruited and detailed information has been collected on these women and their offspring at regular intervals. The study website contains details of all the data that are available through a fully searchable data dictionary (http://www.bris.ac.uk/alspac/researchers/data-access/data-dictionary/). Written informed consent has been obtained for all ALSPAC participants. Ethics approval for the study was obtained from the ALSPAC Ethics and Law Committee and the Local Research Ethics Committees. For this study, we used data from the ARIES (25), a subsample of ALSPAC on whom genome-wide DNA methylation data has been collected at multiple time points.

### Smoking variables

Information on smoking behaviour for the ARIES cohort came from questionnaires completed at the median ages 14 years 2 months, 15 years 5 months and 16 years 7 months (hereafter referred to as 14 years, 15 years and 16 years).

#### Ever smoking

A question on ever smoking was asked in all three questionnaires up to and including age 16 years and used to derive variable for ‘ever smoking’ (‘yes’ or ‘no’). From this variable, we derived a more reliable variable for ‘ever smoking’ by removing those individuals who inconsistently reported their smoking behaviour in the three questionnaires. For example, if an individual reported ever smoking at the first-time point but not at the second or third, they were reported as missing. We used this variable for ever smoking.

#### Current weekly smoking

Current weekly smokers were individuals who reported smoking at least one cigarette per week at the time the questionnaire was completed at 16 years (being the time point most proximal to when DNA methylation data were obtained). Individuals who reported never having smoked or less than weekly at any of the three questionnaires were defined as less than weekly smokers.

#### Ever weekly smoking

Ever weekly smokers were defined as individuals who reported at least one cigarette smoked weekly at any of the three questionnaires. Less than weekly ever smokers were defined as individuals who had never smoked at least weekly.

#### Blood cotinine levels

Nicotine consumption is commonly measured by cotinine in the blood (50) and in this study blood cotinine levels were assessed in the ALSPAC offspring between ages 15 and 17 and is a continuous variable. Cotinine was assayed from ethylenediaminetetraacetic acid serum plasma samples taken in a clinical assessment. Plasma samples were stored at −80°C and allowed to thaw at room temperature before use. Cotinine was measured using the Cozart Cotinine Enzyme Immunoassay (Concateno UK, Abingdon) serum kit (M155B1). All samples, calibrators and controls were brought to room temperature before use and were run in duplicate. Where required, samples were diluted using cotinine-free serum (foetal calf serum). Absorbance was measured spectrophotometrically at a wavelength of 450 nm.

#### Time since initiation and frequency of smoke exposure

We next investigated patterns of methylation change in relation to duration and frequency of smoke exposure. Two additional variables were derived to investigate time since smoking initiation: time since weekly smoking initiation and time since the individual first ever smoked. Time since weekly smoking initiation was the difference between the age of the individuals at methylation data collection when individuals were aged 17 years and the age the individual began weekly smoking. For this variable, less than weekly smokers were assigned a value of ‘0’.

Time since the individual first ever smoked was the difference between the age of the individuals at methylation data collection when individuals were 17 years and the age the individual first ever tried a cigarette. For this variable, individuals who had never smoked at the mean age 16 years were assigned a ‘0’ value.

To investigate methylation differences with frequency of smoke exposure, we also created a categorical variable based on information obtained from a questionnaire at age 16. The categories were: ‘Never smoked a cigarette’, ‘Only ever smoked cigarettes once’, ‘Used to smoked but doesn’t now’, ‘Sometimes smokes cigarettes but less than once a week’, ‘Smokes between 1 and 6 cigarettes a week’, ‘Smokes more than 6 cigarettes a week’ and ‘Smokes 1 or more cigarettes a day’. For this variable, ‘0’ was defined as ‘No’ for ever smoking at age 16.

Finally, we investigated the effect of cumulative cigarettes ever smoked on DNA methylation, this ordinal variable was obtained from the questionnaire at 16 years 7 months and individuals categorized into ‘Never smoked’, ‘Fewer than 5 cigarettes ever smoked’, ‘Between 5 and 19 cigarettes ever smoked’, ‘Between 20 and 49 cigarettes ever smoked’, ‘Between 50 and 99 cigarettes ever smoked’ and ‘100 or more cigarettes ever smoked’.

### DNA methylation

As part of the ARIES (http://www.ariesepigenomics.org.uk/) project (25), the Illumina Infinium® HM450 BeadChip assay (Illumina, Inc., CA, USA) has been used to generate epigenetic data on 1018 mother–offspring pairs in the ALSPAC cohort. More details about the sample selection and data processing for the ARIES study are available in the Supplementary Methods. For this study we used DNA methylation data from the offspring to investigate associations between self-reported smoking behaviours and DNA methylation in adolescence. Specifically, we investigated methylation β values for 2620 out of 2623 CpGs previously identified in a large-scale EWAS of smoking (10) which were available in the normalized dataset for the ARIES offspring in adolescence (ages 15–17). We also conducted sensitivity analyses where analyses were adjusted for methylation levels at the corresponding CpG sites in childhood (before the onset of smoking) using data from the normalized dataset for the ARIES offspring at age 7.

### Covariates

From previously completed ALSPAC questionnaires we identified the following potential confounders: sex, age, parental social class, smoking by the mother during pregnancy of the offspring for whom methylation is measured, passive smoke exposure, DNA methylation at age 7, regular alcohol consumption and regular cannabis use. The highest parental occupation was used to allocate the children to family social class groups using the 1991 British Office of Population Censuses and Surveys classification. Age was the age recorded at the later DNA methylation collection time point. Smoking by the mother during pregnancy of the offspring for whom methylation is measured was defined as the mother smoking in the first 18 weeks’ gestation. Passive smoke exposure was derived from maternal report of the mother or her partner smoking when the offspring was age 12. Regular alcohol consumption was categorized as alcohol consumed at least once a week or 2 to 4 times a month, depending on the wording of the question, reported by adolescents in any of the three questionnaires. Regular cannabis use was defined as using at least once a week across any of the three questionnaires.

Smoking could rapidly change cell composition which could explain the findings of an immediate impact of smoke exposure on DNA methylation in peripheral blood. To ensure findings were not influenced by variation in cell-type fraction between samples, the fraction of CD8T-, CD4T-, NK- and B-cells, monocytes and granulocytes were estimated based on the methylation data using the *estimateCellCounts* function in the *minfi* Bioconductor package implemented in R (28,29). Models were run adjusted for cell counts and we also investigated associations between ever smoking and current weekly smoking with derived cell counts.

### Statistical analysis

#### Multivariable linear regression

Multivariable linear regression was used to perform association tests between the smoke exposure variables and β values at each CpG site as the outcome. Analyses were run with and without adjustment for several potential confounders found to be previously associated with both smoking status and DNA methylation. All regression analyses were performed in *Stata* (version 15). From the 2620 CpG sites a Bonferroni-corrected threshold of 1.91 × 10^–5^ (0.05/2620) was used to identify ‘EWAS-significant’ hits where association *P*-values below this threshold were considered a likely true positive, worthy of further examination.

Four models for the regression analysis for the own smoking variables were examined:

- Basic Model: Adjusted for batch and cell count
- Adjusted Model 1: Adjusted for batch, cell count, age, sex, social class and maternal smoking during pregnancy
- Adjusted Model 2: Adjusted Model 1 covariates, regular cannabis use, regular alcohol use, passive smoke and maternal smoking during pregnancy
- Adjusted Model 3: Adjusted Model 2 covariates, additionally adjusted for methylation at age 7
- Adjusted Model 4: Adjusted Model 2 covariates, additionally adjusted for cord blood methylation

#### MI of missing data

Not all individuals in the ARIES cohort had complete covariate data; maternal smoking behaviour, maternal social class, regular cannabis use and regular alcohol consumption variables all have missing data. To minimize potential selection bias and improve power in the adjusted models, multivariable MI was used to impute missing data for eligible participants. Missing data MI was performed using the ice command (51) in *Stata* (version 15). This method imputes values for covariates based on patterns for other individuals and other correlated variables. The ice command uses regression switching (52) and for this MI twenty cycles of regression switching were carried out generating 20 imputed datasets. Full details of the MI models are outlined in the Supplementary Methods.

#### Time since initiation and frequency of smoke exposure

In this analysis, we investigated differences in methylation levels between each of the smoking categories compared with the reference group of never smokers using indicator variables. Models were run adjusted for batch, cell count, age, sex, social class and maternal smoking during pregnancy. Results were presented in a heatmap dendrogram generated in *R* (version 3.2.2) using a hierarchical clustering approach to group sites with similar patterns of methylation change.

#### Longitudinal latent class analysis

LLCA was performed using *Mplus v8* (26) to derive smoking classes based on a four-category ordinal variable with categories ‘none’, ‘less than weekly’, ‘weekly’ and ‘daily’ smoking from the three questionnaire time points (14, 15 and 16 years). LLCA assumes that variability in response is due to a latent (unobserved) grouping. Starting with a single class, a series of models were fitted and theoretical and statistical steps were taken to decide on the optimal number of latent classes, for further detail on the derivation of the smoking latent classes, see Heron *et al*. (24). The LLCA was applied to ARIES participants who had information on smoking frequency available at the three time points. The regression analysis was performed in *Stata*, adjusting for batch, cell count, age, sex, social class and maternal smoking during pregnancy.

#### Interaction analysis

The 2620 CpG sites associated own smoking were compared to 568 CpG sites which surpassed a Bonferroni-correction threshold in a large EWAS of maternal smoking during pregnancy and cord blood DNA methylation (27). There were 119 CpG sites overlapping in both sets of CpG sites (Table S7). Using these sites, interaction analysis was performed for each of the four own smoking measures. Multivariable regression analysis was performed using covariates outlined in Model 1 (batch, cell count, age, sex, social class and maternal smoking during pregnancy) along with an interaction term for maternal smoking during pregnancy and the own smoking measure.

## Supplementary Material

Supplementary Data
