# Supplementary material for: Investigating the impact of cigarette smoking behaviours on DNA methylation patterns in adolescence
Source: Hum Mol Genet. 2018 Sep 12;28(1):155–65. doi: 10.1093/hmg/ddy316 (PMC6298233; doi:10.1093/hmg/ddy316)
Supplement: Supplementary Data [file ddy316_supp.zip › Supplementary data.docx]

Supplementary Figure 1 Smoking behaviour profiles from four-class model

*
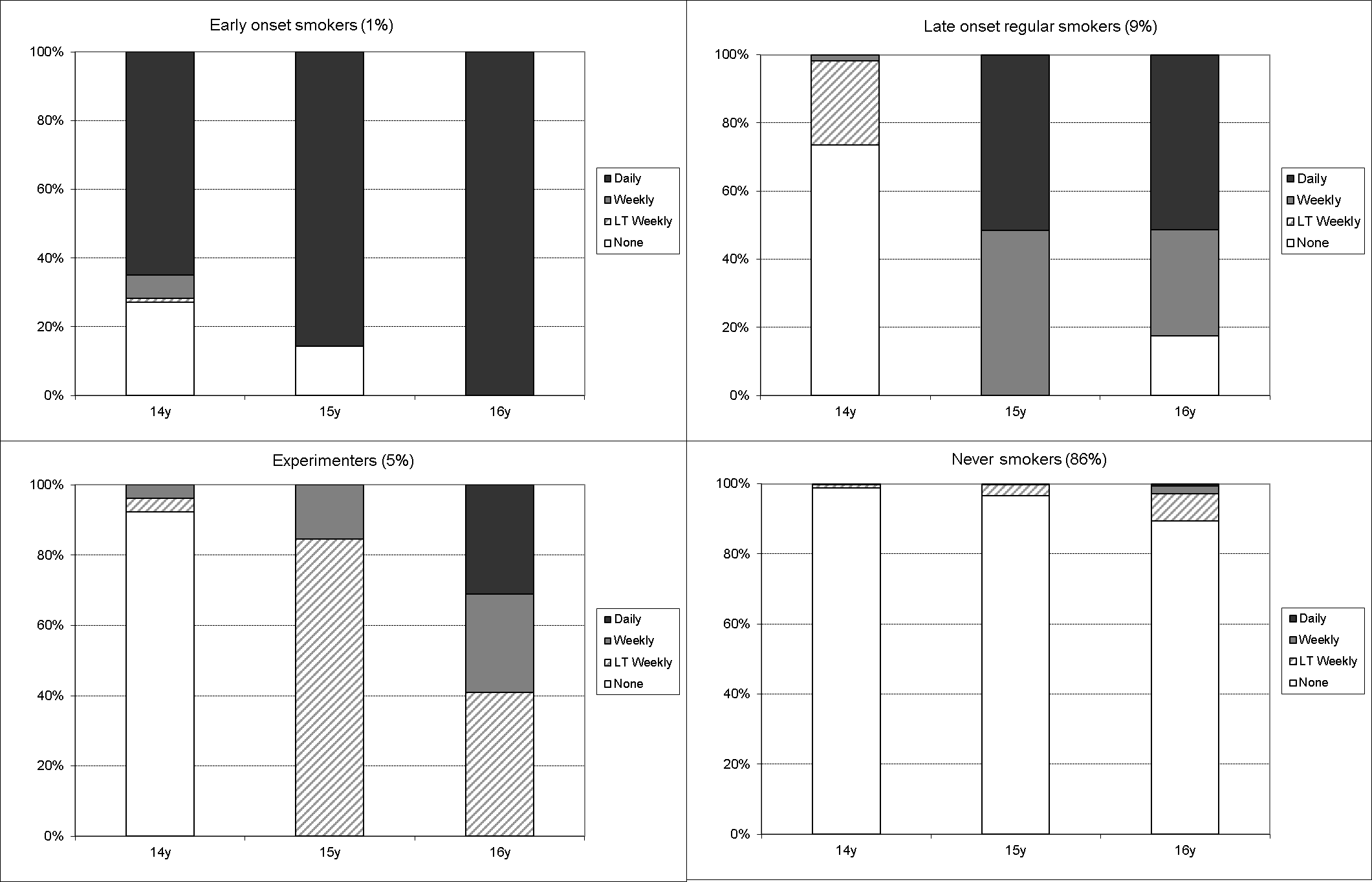
*

*
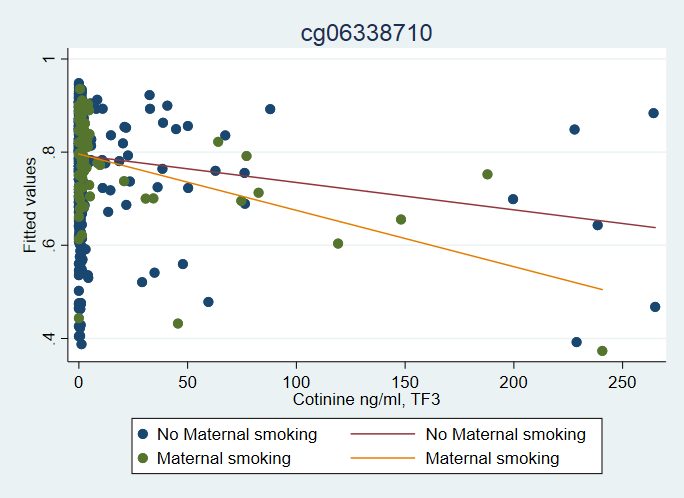
Supplementary Figure 2 cg06338710 (*GFI1) *methylation interaction between own smoking and maternal smoking in adolescence.*

All Supplementary Tables can be found in a separate excel document.

**Supplementary Methods**

**DNA methylation**

The ARIES participants were selected based on availability of DNA samples at two-time points for the mother (antenatal and at follow-up when the offspring was in adolescence) and at three-time points for the offspring [neonatal, childhood (age 7) and adolescence (age 17)].

The DNA methylation wet laboratory and pre-processing analyses were performed at the University of Bristol as part of the ARIES project. Following DNA extraction, samples were bisulphite-converted using the Zymo EZ DNA MethylationTM kit (Zymo, Irvine, CA, USA). Following conversion, genome-wide methylation status of over 485 000 CpG sites was measured using the Infinium HM450 BeadChip according to the standard protocol. The arrays were scanned using an Illumina iScan and initial quality review was assessed using GenomeStudio (version 2011.1). Samples from all time points in ARIES were distributed across slides using a semi-random approach (sampling criteria were in place to ensure that all time points were represented on each array) to minimize the possibility of confounding by batch effects. In addition, during the data generation process a wide range of batch variables were recorded in a purpose-built laboratory information management system (LIMS). The main batch variable was found to be the bisulphite conversion (BCD) plate number. Samples were converted in batches of 48 samples and each batch identified by a plate number. The LIMS also reported QC metrics from the standard control probes on the 450K BeadChip for each sample.

Sample quality control (QC) and normalisation was completed using *meffil* in R version 3.2.0. Methylation quality was check by: genotype QC (sample swaps, gender mismatches, high IBD or relatedness issues), the median intensity methylated vs unmethylated signal for all control probes, dye bias, detection p value, low bead numbers and post normalisation checks. Samples were normalised in *meffil*, which involves a re-implementation of function normalisation as implemented in the *minfi* R package and used 10 control probe principal components derived from technical probes. (30)

The Infinium HM450 BeadChip assay detects the proportion of molecules methylated at each CpG site on the array. For the samples, the methylation level at each CpG site was calculated as a beta value (β), which is the ratio of the methylated probe intensity and the overall intensity and ranges from 0 (no cytosine methylated) to 1 (complete cytosine methylated) (31, 32).

**Multiple Imputation Methods**

87.9% of individuals with information on ever smoking have data on maternal social class data. 94.6% of individuals with information on ever smoking have data on maternal smoking during pregnancy. 97.6% of individuals with information on ever smoking have data on whether they drink alcohol regularly or not. 98.7% of individuals with information on ever smoking have data on whether they use cannabis regularly or not. **Supplementary Table 2** presents each variable included in the imputation model smoke exposure variables, CpG site variables, potential confounding variables and possible predictors of the missing data), the type of variable (i.e. continuous, binary or categorical) and the type of regression model used to predict missing data in this variable. The multiple multivariable imputation approach creates a specified number of copies of the data (20 copies) in which missing values are imputed by chained equations, with an appropriate level of randomness. The main results presented in this paper on the multiple imputation datasets are obtained by averaging the results from each of these 20 datasets using Rubin’s rules. In this procedure, the standard errors for any regression coefficients (used to calculate p-values and 95% confidence intervals) take account of the uncertainty in the imputations as well as uncertainty in the estimate. There was no substantial collinearity between variables included in the multiple imputation models.

**Time since initiation and frequency of smoke exposure heatmap**

Heatmaps (**Figures 2a-2d)** produced using R software which allows clustering analysis; the grouping of, in this case, CpG sites with similar patterns across the number of years smoking. The “Color key” represents the change in the β value of methylation of each CpG site between years or intensities and the reference group (no smoke exposure).
